# Classification of adults suffering from typical gastroesophageal reflux disease symptoms: contribution of latent class analysis in a European observational study

**DOI:** 10.1186/1471-230X-14-112

**Published:** 2014-06-26

**Authors:** Stanislas Bruley des Varannes, Renzo Cestari, Liudmila Usova, Konstantinos Triantafyllou, Angel Alvarez Sanchez, Sofia Keim, Paul Bergmans, Silvia Marelli, Esther Grahl, Philippe Ducrotté

**Affiliations:** 1 Institut des Maladies de l’Appareil Digestif – CHU Hôtel Dieu, 44093 Nantes Cedex, France; 2 Università degli Studi, Spedali Civili, Brescia, Italy; 3 Regional Clinic Hospital, Nizhny Novgorod, Russia; 4 University General Hospital Attikon, Athens, Greece; 5 Hospital Clinico San Carlos, Madrid, Spain; 6 Janssen-Cilag, Barcarena, Portugal; 7 Janssen-Cilag B.V., Tilburg, The Netherlands; 8 Janssen-Cilag, Milan, Italy; 9 Janssen-Cilag, Neuss, Germany; 10 CHU Rouen, Rouen, France

**Keywords:** GERD, Acid related disease, Adults, Latent class analysis, Symptoms, Classification

## Abstract

**Background:**

As illustrated by the Montreal classification, gastroesophageal reflux disease (GERD) is much more than heartburn and patients constitute a heterogeneous group. Understanding if links exist between patients’ characteristics and GERD symptoms, and classify subjects based on symptom-profile could help to better understand, diagnose, and treat GERD. The aim of this study was to identify distinct classes of GERD patients according to symptom profiles, using a specific statistical tool: Latent class analysis.

**Methods:**

An observational single-visit study was conducted in 5 European countries in 7700 adults with typical symptoms. A latent class analysis was performed to identify “latent classes” and was applied to 12 indicator symptoms.

**Results:**

On 7434 subjects with non-missing indicators, latent class analysis yielded 5 latent classes. Class 1 grouped the highest severity of typical GERD symptoms during day and night, more digestive and non-digestive GERD symptoms, and bad sleep quality. Class 3 represented less frequent and less severe digestive and non-digestive GERD symptoms, and better sleep quality than in class 1. In class 2, only typical GERD symptoms at night occurred. Classes 4 and 5 represented daytime and nighttime regurgitation. In class 4, heartburn was also identified and more atypical digestive symptoms. Multinomial logistic regression showed that country, age, sex, smoking, alcohol use, low-fat diet, waist circumference, recent weight gain (>5 kg), elevated triglycerides, metabolic syndrome, and medical GERD treatment had a significant effect on latent classes.

**Conclusion:**

Latent class analysis classified GERD patients based on symptom profiles which related to patients’ characteristics. Although further studies considering these proposed classes have to be conducted to determine the reproducibility of this classification, this new tool might contribute in better management and follow-up of patients with GERD.

## Background

Gastroesophageal reflux disease (GERD) refers to reflux of the gastric content into the esophagus, leading to esophagitis, reflux symptoms impairing the quality of life, or long-term complications [1].

GERD is one of the most common disorders seen by primary care providers and gastroenterologists in the United States as well as in Europe [2]. Besides the millions of medical visits to physicians and the billions of dollars in annual costs [3], frequent or severe GERD symptoms are associated with time lost from work [4], impaired health-related quality of life [5], and esophageal adenocarcinoma [6], further emphasizing the clinical significance of this entity.

Although heartburn is highly specific to characterize GERD, patients usually report a number of associated symptoms such as regurgitation, nausea, sore throat, cough, eructation, globus, hiccups, chest pain, sleep disturbances, etc [7,8]. Consequently, individuals with the typical GERD symptoms of heartburn and acid regurgitation constitute a heterogeneous group. The combinations of the different symptoms, their associations, but also their link with clinical or demographic factors (age, body mass index [BMI], history of GERD, comorbidities, etc.), might be more prominent in certain groups of patients. An improvement in the understanding of the structure underlying these individual differences in GERD symptoms would help to individualize subject profiles and to adapt GERD management.

The difficulty is to find tools organizing the many symptoms and identify groups of patients suffering from a specific combination of symptoms. A statistical method that could help making such distinctions is the latent class analysis (LCA). LCA can best be thought of as an “improved” cluster analysis, which uses statistical (rather than mathematical) methodology to construct the results. It is based on the statistical concept of likelihood. Parameters are estimated for class profiles (the description of each class) and the size of each class. A difference is that cases are not absolutely assigned to classes, but have a probability of membership for each class. It can deal with all types of data – binary, continuous and count data. So, first of all, the aim of a LCA is to reduce the complexity of the data by identifying clusters of observed variables, the latent classes, and then, to reduce the heterogeneity of the population of patients by identifying sub populations based on the probability of each of the subjects to belong to a certain latent class. Compared with cluster analysis applied to ordinal or continuous variables, a LCA can also be performed to categorize people on dichotomous variables and can quantify the extent to which indicators are not perfectly related to class membership (i.e., measurement error) [9]. The resulting latent class represents the groups of homogeneous individuals within the class to which they belong. These groups are then heterogeneous across the different classes.

Recently, the LCA has been used in various health conditions to examine subgroups in weight loss strategies used among women [9], parenting characteristics associated with children’s BMI [10], obesity risk [11], and maternal pregnancy weight status associated with Attention Deficit/Hyperactivity Disorder in their offspring [12]. The results of these different studies have shown that the LCA is an effective and valid method to categorize individuals with similar characteristics.

The purpose of this study was to try to identify distinct classes of adult subjects with respect to their different GERD symptoms by means of a LCA, and to investigate if there is a relation between these latent classes and associated factors.

## Methods

### Study design

This international, multicenter, non-interventional, observational study was conducted in France, Greece, Italy, Russia, and Spain. A total of 7917 adult subjects suffering from GERD and presenting at least one typical GERD symptom (i.e., heartburn and/or regurgitation), at least once a week, in the week prior to the first (and only) study visit, were enrolled in the study. As this was a non-interventional study, neither changes to the current treatment that the subject received, were required, nor was additional treatment provided by the Sponsor.

This study was conducted in accordance with the ethical principles that have their origin in the Declaration of Helsinki and was consistent with the applicable regulatory requirements. Local ethics committee approval was obtained where required (see Additional file 1 – Study approvals), and all participants provided written informed consent (except for France where it was not required for this type of data collection).

### Participants

Participants were men or women consulting their general practitioner or a specialist, at least 18 years old, who had an established diagnosis of GERD according to the investigator, or an occurrence of at least one typical GERD symptom (i.e., heartburn or regurgitation), at least once a week in the last 3 months, and presented at least 1 of the typical GERD symptoms (i.e., heartburn or regurgitation) in the week preceding the study visit. Subjects who exclusively presented atypical digestive or non-digestive symptoms (e.g., epigastralgia, respiratory disturbances, thoracic manifestations, etc.), had a current or recent (less than 1 year) history of gastric or duodenal ulcer, a history of surgery of the upper digestive tract, or a tumor of the superior digestive tract or ears-nose-throat (ENT) system were not selected. The study was performed by specialty sites (gastroenterologists, endoscopists, and internal medicine specialists) in all participating countries, except for France, where it was performed by general practitioners.

All subjects documented in this study continued to receive treatment as required, according to usual care in their treatment setting and at the discretion of the treating physician.

### Assessments

At the single visit, the subject’s characteristics of the participants were recorded (demographic data, medical history) as well as all available data about comorbidities (diabetes, metabolic syndrome, cardiovascular diseases, osteoarthropathic treatments, irritable bowel syndrome, sleep apnea, and anti-reflux and bariatric surgery), concomitant medication, smoking, drinking, and other lifestyle habits, as well as if the main purpose of the participant’s visit was GERD.

Weight, height, and waist circumference (using a tape measure) were measured, and BMI was calculated. The investigator also asked if the subject remembered his/her weight from 12 and 24 months ago, and the weight change in the last 12 months was recorded.

The typical GERD symptoms (heartburn and regurgitation), present in the week before the first (and only) study visit, were recorded in detail by means of specific multiple-choice questions regarding time (daytime and/or nighttime), severity and frequency of occurrence, and the overall evolution of GERD symptoms in the last 12 months. Atypical digestive (nausea, eructation, slow digestion/early satiety, epigastralgia) and non-digestive (thoracic manifestations i.e., atypical precordial pain, respiratory disturbances i.e., cough, and ENT symptoms i.e., hoarseness, pharyngeal pain, globus, etc.) GERD symptoms, present in the week before the study visit, were also documented as well as “warning signs”, such as dysphagia, weight loss, anemia, anorexia, gastrointestinal bleeding, asthenia, and vomiting. As nighttime GERD can cause sleep disorders and thus affect quality of life, each subject was asked to evaluate his/her quality of sleep and to report the presence of sleep disturbances (early awakening, difficulty falling asleep, nocturnal awakening, nightmares) in the week before the study visit.

Finally, the investigator documented the subject’s GERD history (first diagnosis of GERD as well as the occurrence of a previous endoscopy), GERD management (including data about GERD non-medical and medical treatment), as well as recommendations for future GERD consultations (if any).

The latent class analysis was performed on 13 indicator variables (i.e., daytime heartburn, daytime regurgitation, nighttime heartburn and/or nighttime regurgitation, nausea/eructation, slow digestion/early satiety, epigastralgia, dysphagia/vomiting, digestive bleeding/anemia, thoracic manifestations, respiratory disturbances, ENT symptoms, early awakening/difficulty falling asleep, nocturnal awakening/nightmares), described as signs and symptoms characterizing GERD.

### Statistical analysis

#### Datasets

The “per-protocol” set of subjects was defined as all subjects who met all inclusion/exclusion criteria. The primary analysis (i.e., latent class analysis, LCA), was performed on all subjects from the per-protocol analysis set with non-missing indicator variables.

#### Latent class analysis

The classification of subjects was carried out by performing a LCA [13-16] on 13 binomial (present/absent) indicator variables representing 13 indicator symptoms. Multiple LCA models were explored with the number of classes set to 2, 3, 4, 5, 6, 7, 8, 9, 10, and 11. The optimal number of classes came from the model with the best model fit.

The descriptive analysis of nominal/ordinal data comprised tabulation of frequency and percentages. The descriptive analysis of continuous data comprised the mean, standard deviation, median, extreme values, and 95% confidence interval. Statistical comparisons between groups, if appropriate, were performed using the Fisher’s exact test (nominal data) and the Wilcoxon two-sample test (ordinal/continuous data). Multinomial logistic regression modeling was used to explore the relation between the classification and associated factors.

All analyses were performed using Statistical Analysis Software (SAS version 9.1), except for the LCA, which was performed with Mplus (version 6.11).

#### Sample size determination

In this type of exploratory study with the chosen method of analysis, a large sample was typically required in order to adequately investigate the objectives. Based on the large number of variables of interest and the availability of resources, the sample size was planned to be 8200. The possible number of symptom profiles was equal to 8192 (2 power 13). If the sample size was equal to or larger than 8192, theoretically, each symptom profile could possibly be represented at least once. Such a sample size would avoid obtaining a sparsely populated multivariate contingency table, thereby reducing the validity of the likelihood ratio statistics for setting the number of latent classes.

## Results

### Participants

Overall, 7917 subjects, enrolled in five countries (France, Greece, Italy, Russia, and Spain), participated in this single-visit observational study. The per-protocol population consisted of 7700 subjects and the primary analysis was performed on 7434 subjects. The subject’s overall characteristics can be found in Additional file 2. Although the primary analysis set was less than the calculated sample size, the representativeness of the effective sample was not affected, because the sample size was based on the theoretically possible number of unique symptom profiles and the included subjects presented approximately 1400 different profiles.

### Latent class analysis

LCA is a statistical method to identify unobserved subgroups of related cases (latent classes) from a set of observed categorical variables. For this study the categorical data that were used were 13 typical GERD symptoms. The LCA model parameters were the prevalence of each of the latent classes and conditional response probabilities such as, the probability for each patient of being member of each class and for each of the categorical variables the probability for each latent class. Parameters were estimated by the maximum likelihood criterion. The LCA was performed on 7434 subjects (only the ones with non-missing indicator variables were included). Of the 7434 subjects included in the primary analysis set, 124 had a positive score on indicator variable S8 (digestive bleeding/anemia). Because this indicator variable showed a very low probability (<0.1) in all classes in all LCA models that were investigated, it was decided to exclude this indicator variable from the analysis and to re-run the LCA on 12 indicator variables.

A stepwise approach was used to determine the optimum number of classes meaning that the LCA was run multiple times with the number of classes set to 2, 3, 4, 5, 6, 7, 8, 9, 10 and 11. The Bayesian information criterion (BIC), the Lo-Mendell-Rubin test, the Akaike information criterion (AIC) and the likelihood ratio chi-square were used to assess the number of latent classes. These statistical parameters did not unequivocally yield one optimal number of classes but indicated solutions of 5, 6 or 7 classes. Therefore, clinicians involved in the protocol scientific committee were asked which classification (5, 6 or 7 classes) was the most conform to clinical experience.

At the end, the 5-class classification was preferred because it yielded clinically well recognizable class-profiles and the interpretation of a 5-class classification was easier than classifications with more classes. Each of these 5 latent classes had its own characteristic probability profile for the 12 indicator symptoms. Table 1 shows the number of subjects with GERD indicator symptoms that were used in the LCA.

**Table 1:** Number of subjects for each indicator variable (primary efficacy set N = 7434)

	**n (%)**
S1: daytime heartburn	6319 (85.0)
S2: daytime regurgitation	5217 (70.2)
S3: nighttime heartburn and/or nighttime regurgitation	4540 (61.1)
S4: nausea, eructation	3852 (51.8)
S5: slow digestion, early satiety	2758 (37.1)
S6: epigastralgia	3154 (42.4)
S7: dysphagia, vomiting	1169 (15.7)
S8: digestive bleeding, anemia*	124 (1.7)
S9: thoracic manifestations (atypical precordial pain)	1125 (15.1)
S10: respiratory disturbances (cough)	1522 (20.5)
S11: ENT symptoms (hoarseness, pharyngeal pain, globus, etc.)	1783 (24.0)
S12: early awakening, difficulty falling asleep	2003 (26.9)
S13: nocturnal awakening, nightmares	2799 (37.7)

n = number of subjects with observations.

ENT = ears, nose, throat.

*Indicator variable S8 (digestive bleeding, anemia) was not included in the latent class analysis.

#### Probability profile for the 12 GERD indicator symptoms

**Figure 1:**
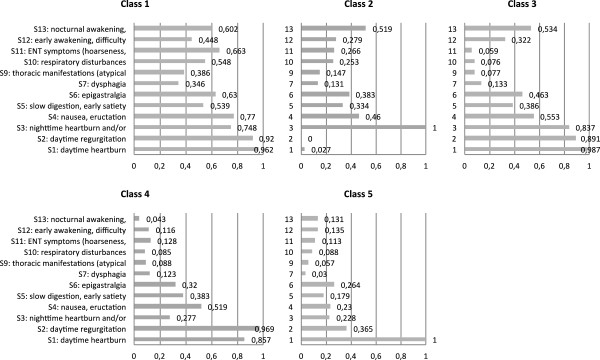
**Probability profile for a positive score on the indicator symptoms for each of the 5 latent classes (primary analysis set N = 7434).** S8 is not presented in the Figure because, due to its very low probability, it was not included in the final LCA. On the X-axis the probability is reported.

The indicator symptom probability profile for each of the 5 classes is presented in Figure 1.

Each class of subjects was described using typical and atypical GERD symptoms, digestive and non-digestive symptoms, warning signs, and sleep disturbances.

Class 1 (N = 1598, 21.5%) represented subjects with a very high probability of daytime heartburn and regurgitation, and a high probability of nighttime heartburn and/or regurgitation. The probability of all other symptoms was higher than in any of the other classes.

Class 2 (N = 845, 11.4%) was characterized by the absence of heartburn and regurgitation during the day and presence of the typical symptoms during the night. Nighttime problems were also reflected by a medium probability of nocturnal awakenings and nightmares.

Class 3 (N = 2375, 31.9%) also showed a high probability of daytime as well as nighttime heartburn and regurgitation. A medium probability was seen for atypical digestive symptoms (S4-S6) and for sleep disorders. Class 3 differed from class 1 in the low probability of non-digestive symptoms (S9-S11).

Class 4 (N = 1181, 15.9%) was the third class with a very high probability of daytime heartburn and regurgitation, however, the probability of nighttime heartburn or regurgitation was low. The probability of the other symptoms was comparable to class 3, except for nocturnal awakening and nightmares. The low probability of these latter symptoms corresponded to the absence of the nighttime core symptoms.

Class 5 (N = 1435, 19.3%) was different from the other classes because in this class there was only one symptom with a high probability: daytime heartburn. Daytime regurgitation had a medium probability and the probability of all other symptoms was low.

#### Comparison among the 5 latent classes

**Figure 2:**
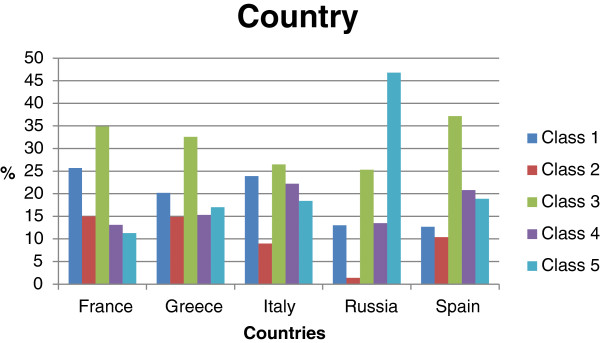
Country distribution per class (primary analysis set N = 7434).

The relative distribution of the 5 classes per country is shown in Figure 2.

More than 45% of the Russian subjects were found in class 5 and very few of them in class 2. For the other countries, less than 20% of subjects were in class 2. There were less French subjects in classes 4 and 5, than in classes 1, 2, and 3.

The gender distribution was equal in all classes except for class 1 where 57% were females.

**Figure 3:**
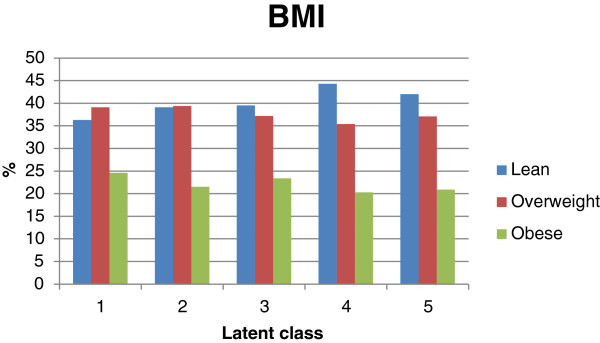
BMI distribution per class (primary analysis set N = 7434).

BMI did not differ between classes. Number of overweight/obese subjects varied from 56% in class 4 to 64% in class 1 (Figure 3). Based on the subject’s history data, the highest occurrences of medical history or concurrent comorbidities were found in class 1 (see Additional file 2).

**Figure 4:**
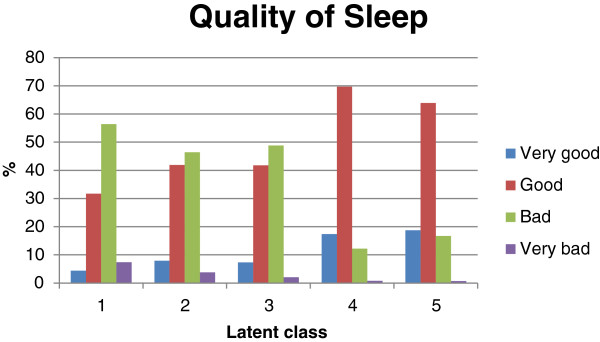
Quality of sleep per class (primary analysis set N = 7434).

The frequency table of the typical GERD symptoms for each class is presented in Table 2 while the atypical digestive as well as non-digestive GERD symptoms are described in Table 3. Warning signs were mostly applicable for the subjects in class 1 (46.2%) and dysphagia was the most commonly reported warning sign (29.6% of the subjects in class 1).In classes 1, 2, and 3, most subjects (85.1, 73.5, and 70.7%, respectively) had sleep disorders; hence, they considered their quality of sleep mainly as bad or very bad (Figure 4). Compared to the other classes, all sleep disorder-related symptoms were reported more frequently in class 1.

**Table 2:** Typical GERD symptoms in the 5 classes (primary efficacy set N = 7434)

**n (%)**	**Class 1**	**Class 2**	**Class 3**	**Class 4**	**Class 5**
	**N = 1598**	**N = 845**	**N = 2375**	**N = 1181**	**N = 1435**
**Heartburn**					
None	53 (3.3)	41 (4.9)	12 (0.5)	204 (17.3)	0
Mild	325 (20.3)	310 (36.7)	599 (25.2)	369 (31.2)	684 (47.7)
Moderate	913 (57.1)	423 (50.1)	1439 (60.6)	544 (46.1)	681 (47.5)
Severe	307 (19.2)	71 (8.4)	325 (13.7)	64 (5.4)	70 (4.9)
**Regurgitation**					
None	107 (6.7)	157 (18.6)	199 (8.4)	2 (0.2)	1063 (74.1)
Mild	543 (34.0)	356 (42.1)	989 (41.6)	650 (55.0)	225 (15.7)
Moderate	757 (47.4)	273 (32.3)	1031 (43.4)	465 (39.4)	130 (9.1)
Severe	191 (12.0)	59 (7.0)	156 (6.6)	64 (5.4)	17 (1.2)
**Number of days per week**					
1 day per week	70 (4.4)	171 (20.2)	131 (5.5)	159 (13.5)	258 (18.0)
2 to 3 days per week	529 (33.1)	407 (48.2)	823 (34.7)	523 (44.3)	631 (44.0)
4 to 5 days per week	546 (34.2)	171 (20.2)	804 (33.9)	279 (23.6)	309 (21.5)
6 to 7 days per week	453 (28.3)	96 (11.4)	617 (26.0)	220 (18.6)	237 (16.5)
**Time of occurrence**					
Only during the day	405 (25.3)	0	189 (8.0)	1094 (92.6)	1206 (84.0)
Only during the night	1 (0.1)	845 (100)	0	0	0
Both during day and night	1192 (74.6)	0	2186 (92.0)	87 (7.4)	229 (16.0)

N = number of subjects.

n = number of subjects with observations.

**Table 3:** Occurrence of atypical GERD Symptoms in the 5 classes (primary efficacy set N = 7434)

	**Class 1**	**Class 2**	**Class 3**	**Class 4**	**Class 5**
**n (%)**	**N = 1598**	**N = 845**	**N = 2375**	**N = 1181**	**N = 1435**
**Digestive GERD symptoms**					
Reported any atypical digestive symptom*	1536 (96.1)	656 (77.6)	1972 (83.0)	1097 (92.9)	652 (45.4)
Slow digestion/early satiety	880 (55.1)	285 (33.7)	874 (36.8)	516 (43.7)	203 (14.1)
Other digestive symptoms	197 (12.3)	75 (8.9)	208 (8.8)	114 (9.7)	169 (11.8)
Epigastralgia	1004 (62.8)	326 (38.6)	1045 (44.0)	474 (40.1)	305 (21.3)
Eructation	1037 (64.9)	331 (39.2)	1042 (43.9)	566 (47.9)	147 (10.2)
Nausea	508 (31.8)	125 (14.8)	410 (17.3)	217 (18.4)	125 (8.7)
**Non-Digestive GERD symptoms**					
Thoracic manifestations^1^	643 (40.3)	127 (15.0)	168 (7.1)	116 (9.8)	71 (5.0)
Pulmonary symptoms^2^	971 (60.8)	216 (25.6)	138 (5.8)	74 (6.3)	123 (8.6)
ENT symptoms^3^	1181 (73.9)	227 (26.9)	40 (1.7)	168 (14.2)	167 (11.7)

N = number of subjects.

n = number of subjects with observations.

ENT = ears, nose, throat.

*Subjects could report more than one atypical digestive symptom.

^1^Atypical precordial pain.

^2^Cough, etc.

^3^Hoarseness, pharyngeal pain, globus, etc.

#### Relation between the classification in the 5 latent classes and subject- and GERD-related factors

The relation between the classification in 5 latent classes and subject- and GERD-related factors was explored using multinomial logistic regression modeling. The LCA classes were considered as dependent binomial variables without any ordering of the latent classes.

In total 24 different factors were examined. Of the factors that were found highly correlated, the one with the strongest effect was kept in the final model. The following 11 factors were entered into this final model:

–The factor with the strongest effect was *country*.

–For the factors *gender, smoking,* and *alcohol use*, a difference was seen between class 1 and the other classes. Class 1 was the only class where females (57%) and males (43%) were not equally represented. Class 1 represented the highest percentage of smoking subjects (31%), which varied from 22 to 28% in the other classes. Alcohol use was also highest in class 1 (42%), this varied from 36 to 38% in the other classes.

–Although *weight and BMI* did not show an effect in the model, the factors *recent weight loss* and *weight gain >5 kg* did. The number of subjects with recent weight loss was highest in class 1 (34%) and lowest in class 5 (24%). Weight gain >5 kg occurred less frequently and varied from 4 to 6% (classes 1 and 5). *Waist circumference* was also highest in class 1 but the differences were small.

–A *low fat diet* was adopted by 58% of the subjects in classes 4 and 5. In the other classes this varied from 46 to 54% (classes 2 and 1, respectively).

–*Metabolic syndrome* was observed in 20% of the subjects of class 1 and varied from 11% in class 5 to 15% in class 2. Highest percentage of *elevated triglycerides* was in seen class 3 (66%); in class 1 this was 63%, and the lowest in class 5 (54%)

–*Medical GERD treatment* was most reported in classes 1 (69%) and 2 (68%), and fewest in classes 4 and 5 (both 62%).

## Discussion

Using a LCA based on 12 indicator variables, representing typical and atypical symptoms of GERD, warning signs, and sleep disturbances, 5 symptom profile classes were identified in 7434 European GERD patients. Because no statistical tool allows to determine the optimal number of classes that mirror real life, the final decision to go for a classification in 5 classes (rather than in 6 or 7 classes) was taken according to an expert-investigators’consensus. This consensus was based, on one side, on clinical experience and practice and, on the other side, on practical considerations as using a relatively low number of classes (i.e. 5 classes) when a relatively limited number of indicators (i.e. 12 indicators) are evaluated. Our opinion is that this approach will increase the probability for clinicians to appropriate themselves such a classification; of course, the relevance of the classification in 5 classes should be prospectively tested in usual care conditions.

Class 1 (very high probability of daytime regurgitation and heartburn; high probability of nighttime typical symptoms and all other symptoms as well) comprised patients where the typical GERD symptoms had the highest severity. Patients also had more digestive as well as non-digestive GERD symptoms and their sleep quality was worse than in the other classes. This class grouped patients with the worst symptoms overall. In this class, almost half of the patients in PPI therapy took them continuously. Class 3 (high probability of daytime and nighttime heartburn and regurgitation; low probability of non-digestive symptoms) was quite comparable to class 1 with patients with a high probability and severity of typical symptoms, occurring during the day as well as the night. As in class 1, almost half of the patients who took PPIs, took them continuously. However, the digestive GERD symptoms and the sleep disturbances occurred less and were less severe than in class 1. The main difference between class 1 and 3 was the probability of non–digestive symptoms that was low in class 3. Class 2 (very high probability of nighttime typical symptoms; no daytime heartburn and regurgitation) differed from the other classes, mainly in the fact that patients only suffered from typical symptoms during the night. Although a comparable PPI treatment modality was observed (i.e., almost half of the PPI users took this treatment continuously), heartburn and regurgitation were less severe than in class 1 and 3. Classes 4 and 5 (high probability of daytime heartburn; low probability of nighttime heartburn and/or regurgitation) showed a quite similar patients’ profile, except for daytime regurgitation that was present with high probability only in class 4. The frequency of typical GERD symptoms was lower than in classes 1 and 3 and a little higher than in class 2. The severity of heartburn was lower in classes 4 and 5 than in the other classes but still moderate/severe for half of the subjects. Class 5 (only a high probability of daytime heartburn) differed from class 4 mainly in the severity of regurgitation: moderate to severe for only approximately 10% of the subjects compared to almost 45% in class 4. Also the occurrence of atypical digestive symptoms was higher in class 4. PPI use was somewhat lower in class 5 than in class 4, but with the same treatment modality (i.e., continuous treatment) of the other classes.

In our study, minor differences among the 5 classes were seen in demographic data (i.e., the percentage of overweight/obese subjects was highest in class 1, but the differences were small), while the factor with the strongest effect on patients’ distribution was the country. In Russia 47% of the subjects was found in class 5, mainly suffering from daytime heartburn as the only symptom with a high probability. Class 1 represented the subjects with the highest probability of all symptoms; there were twice as much French, Greek, and Italian subjects in this class than Spanish and Russian. Class 3, with less digestive and non-digestive symptoms than in class 1, was most represented in the Spanish population. These results illustrate the likely country-specific expression of symptoms for the same disease. Additional studies are requested to determine whether these differences are linked to relative pathophysiological differences or only to cultural characteristics in expressing symptoms.

Although the applicability and relevance of these 5 classes have now to be established in clinical practice, this approach may be of interest in GERD. In fact, reflux symptoms are common, especially in primary care, but definition and classification of GERD remain uneasy due to its wide spectrum of clinical manifestations. Most patients with GERD do not have erosive disease, are labeled as having NERD, but they can experience GERD symptoms just as severely as do patients with endoscopically confirmed mucosal damages [17]. For this reason, symptom evaluation is a crucial aspect both in the diagnosis of GERD (i.e., when objective tests as endoscopy and oesophageal pH evaluations are relatively insensitive) and in the evaluation of the effectiveness of therapy, when a diagnosis of GERD has been already established. Heartburn and acid regurgitation have long been considered as the cardinal symptoms of GERD and have been necessary inclusion criteria in most therapeutic trials. But the clinical manifestations of GERD are now appreciated to be broad-based, including many atypical and extra-esophageal symptoms such as non-cardiac chest pain and asthma as well as a wide array of associated symptoms such as nausea, lower gastrointestinal complaints, and sleep disturbances that contribute to the marked reduction in quality of life for GERD patients. So, to address patient needs, the full range of GERD symptoms should be taken into account [17,18]. Another element to be considered is that symptoms in addition to/other than heartburn may respond differently to therapy. There are post hoc analyses of studies in endoscopy negative GERD patients which show that reflux symptoms respond less well to PPI therapy in patients who have more non-heartburn symptoms or where heartburn is not dominant. Similarly, regurgitation may not respond as well to therapy as heartburn. Moreover, patients are less likely to respond to initial reflux therapy if they have concomitant symptoms of irritable bowel syndrome and if they are labeled as having NERD [18]. In addition to this, a minority of GERD patients have multiple unexplained symptoms which may be associated with other psychological distress [18].

Recently, the experts who met in Montreal have proposed a symptom subclassification into esophageal and extra-esophageal symptoms [8]. Other investigational teams performed studies to evaluate patients’ symptom profiles in GERD disease. Ponce et al [19]. analyzed the symptom profile of patients with typical GERD manifestations (heartburn and/or regurgitation), comparing untreated patients with those with persistent symptoms despite treatment. A total of 2356 Spanish patients were included in a prospective, observational, cross-sectional study under conditions of standard clinical practice. Dyspeptic symptoms were about 90% in both groups and supra-esophageal ones were also common (50-60%). People with persistent symptoms despite treatment were older, had more supra-esophageal symptoms and the typical and dyspeptic symptoms were more severe in these patients. Older age resulted in a risk factor for supra-esophageal symptoms, female gender for dyspeptic symptoms, and BMI for greater severity of GERD symptoms. In Turkey, Bor et al [20]. performed a study to determine the prevalence and clinical spectrum of GERD in a low-income region. Using a reflux questionnaire validated by Locke et al. at Mayo Clinic, they included 630 randomly selected, low-income participants older than 20 years, living in a population of 8857 Caucasian adults. Their results showed that the prevalence of GERD was similar in low-income populations to that in developed countries, but with a different symptom profile (i.e., a lower incidence of heartburn and a higher incidence of regurgitation and dyspepsia). A comparison between the frequent and the occasional symptom group (i.e., subjects with either of the typical symptoms less than once a week) revealed that only two of the other symptoms, namely dysphagia and odynophagia, were significantly higher in the frequent symptom group. They also found that the prevalence of heartburn symptoms, but not regurgitation, increased significantly with age and that heartburn and regurgitation were both significantly more common in women. In conclusion, both these studies found that dyspeptic symptoms as well as typical and atypical symptoms should all be considered in relation to GERD because they are associated in nearly 50% of patients. Although clinical practice guidelines were closely followed, it seemed that the diagnostic procedures were not always adequate to determine clear different symptom profiles. Thus, our study is an additional attempt to define relevant subgroups in GERD patients, using the LCA.

The LCA applied to eating behavior and physical activity have also provided useful results in determining subtypes of children using multiple dimensions of obesity risks [11] and interesting results have also been reported in weight loss strategies among women [9], parenting characteristics associated with children’s BMI [10], and maternal pregnancy weight associated with attention deficit hyperactivity disorder in their offspring [12].

The results of our LCA have shown that a person-centered analytical approach could be an effective method to determine a classification of adults suffering from typical GERD symptoms and to describe the most frequent symptom profiles and characteristics. Among patients complaining from at least one of the major symptoms of GERD, i.e. heartburn or regurgitation, subgroups exist. This is an important point to be considered for patients’ management in the clinical practice. Indeed, besides heartburn and regurgitation, other symptoms often exist and have to be taken into account in order to provide to the patient an overall and satisfactory symptoms relief. We can speculate that patients'expectations are not the same between, for instance, Class 5 patients suffering only from daytime heartburn and patients from Class 2 or 3 with also nocturnal typical symptoms, a medium probability of atypical symptoms and sleep disorders. Of course, from a clinical perspective, the relevance of this classification should be prospectively tested in further interventional studies and/or in real world setting. These findings may call for refinement of GERD programs, as to our knowledge, this is one of the first attempts in GERD research to estimate latent classes of symptom profiles and characteristics. As this study only comprised one visit, symptom changes over time have not been investigated. Patients could switch classes throughout time due to changes in e.g., therapy, lifestyle habits. However, incorporating these different classes into GERD diagnosis could help in determining a specific patient profile and a specific treatment approach.

## Conclusion

In conclusion, our study has shown, for the first time, the potential interest of using a LCA in determining subgroups of patients with GERD. Further studies considering these proposed classes have now to be conducted to determine the reproducibility of this classification and its contribution in the patient management and follow-up.

## Abbreviations

GERD: Gastroesophageal reflux disease; LCA: Latent class analysis.

## Competing interests

Stanislas Bruley des Varannes and Philippe Ducrotté were consulted as Scientific Committee for the study and therefore received grant support. Renzo Cestari, Liudmila Usova, Konstantinos Triantafyllou, Angel Alvarez Sanchez have received grant support from Janssen for their participation in this study as Investigators. Sofia Keim, Paul Bergmans, Silvia Marelli and Esther Grahl are employees of Janssen.

## Authors’ contributions

SBV, PD: Investigator, study design, manuscript writing. RC, LU, KT, AAS: Investigator. PB: Data management, statistical analysis, manuscript writing. SK, SM, EG: Study design, data management, manuscript writing. All authors have read and approved the final manuscript.

## Pre-publication history

The pre-publication history for this paper can be accessed here:

http://www.biomedcentral.com/1471-230X/14/112/prepub

## Supplementary Material

Additional file 1 **Study approvals.** Click here for file

Additional file 2 Results. Click here for file
