# Supplementary material for: Classification of adults suffering from typical gastroesophageal reflux disease symptoms: contribution of latent class analysis in a European observational study
Source: BMC Gastroenterol. 2014 Jun 26;14:112. doi: 10.1186/1471-230X-14-112 (PMC4094535; doi:10.1186/1471-230X-14-112)
Supplement: Additional file 1 — Study approvals. [file 1471-230X-14-112-S1.docx]

**Additional file 1 – Study approvals**

According to each country’s legislation/requirements for the conduction of observational studies, the following approvals were obtained:

**FRANCE**

Approved by National Research Committee (CCTIRS), Data Privacy Council (CNIL) and National Physician Council (CNOM)

**GREECE**

Approved by each Hospital Scientific Committee

**RUSSIA**

Approved by Central Ethics Committee (FEC) at Federal Service on Surveillance in Healthcare and Social Development - "Roszdravnadzor"

**SPAIN**

Approved by Central Ethics Committee at Hospital Clinico San Carlos, Madrid

**ITALY**

Obtained individual site Ethics Committee approvals as reported below:

| COMITATO ETICO DELLA ASL DI FROSINONE |
| --- |
| COMITATO ETICO PER LA SPERIMENTAZIONE CLINICA DEI MEDICINALI DELLA AUSL 3 DI PISTOIA |
| COMITATO ETICO PER LA SPERIMENTAZIONE CLINICA DEI MEDICINALI DELLA ASL 1 DI MASSA E CARRARA |
| COMITATO ETICO INTERAZIENDALE DELL'AZIENDA OSPEDALIERA S. SALVATORE DI PESARO, DELLA ASUR ZONA TERRITORIALE 1 DI PESARO E ZONA TERRITORIALE 2 DI URBINO |
| COMITATO ETICO SPERIMENTAZIONE CLINICA MEDICINALI DELLA AUSL 8 DI AREZZO |
| COMITATO ETICO DELL'AZIENDA OSPEDALIERA UNIVERSITARIA S. MARTINO DI GENOVA |
| COMITATO ETICO INDIPENDENTE DEGLI OSPEDALI FATEBENEFRATELLI S. GIUSEPPE DI MILANO E SACRA FAMIGLIA DI ERBA (CO) |
| COMITATO ETICO PER LA SPERIMENTAZIONE CLINICA DEI MEDICINALI DELL´AZIENDA SANITARIA DI FIRENZE |
| COMITATO ETICO PER LA SPERIMENTAZIONE DELL'AZIENDA OSPEDALIERA DI PADOVA |
| COMITATO ETICO DELL´OSPEDALE VALDUCE DI COMO |
| COMITATO ETICO PROVINCIALE PER LA SPERIMENTAZIONE CLINICA DI BELLUNO |
| COMITATO ETICO PER LA SPERIMENTAZIONE CLINICA DEI FARMACI DELLA AUSL DI PESCARA |
| COMITATO ETICO DELL'AZIENDA OSPEDALIERA DELLA VALTELLINA E DELLA VALCHIAVENNA DI SONDRIO |
| COMITATO ETICO DELL´AZIENDA OSPEDALIERA S. CROCE E CARLE DI CUNEO |
| COMITATO ETICO PER LA SPERIMENTAIZONE CLINICA DELLA PROVINCIA DI VICENZA |
| COMITATO PER LA SPERIMENTAZIONE CLINICA DEI MEDICINALI DELL´AZIENDA OSPEDALIERO UNIVERSITARIA PISANA DI PISA |
| COMITATO DI ETICA DELLA ASL DI SALERNO |
| COMITATO ETICO DELL'AZIENDA OSPEDALIERA UNIVERSITARIA S. LUIGI GONZAGA DI ORBASSANO (TO) |
| COMITATO ETICO DELL'AZIENDA OSPEDALIERA GUIDO SALVINI DI GARBAGNATE MILANESE (MI) |
| COMITATO ETICO LOCALE PER LA SPERIMENTAZIONE CLINICA DELLA AUSL 12 DI VIAREGGIO |
| COMITATO INDIPENDENTE DI ETICA DELL´AZIENDA OSPEDALIERA CARLO POMA DI MANTOVA |
| COMITATO ETICO DELLA ASL 3 GENOVESE DI GENOVA |
| COMITATO ETICO DELL´AZIENDA OSPEDALIERA ISTITUTI OSPITALIERI DI CREMONA |
| COMITATO ETICO DELL´AZIENDA OSPEDALIERA OSPEDALE DI CIRCOLO E FONDAZIONE MACCHI DI VARESE |
| COMITATO ETICO AZIENDA OSPEDALIERA MONALDI DI NAPOLI |
| COMITATO ETICO DELL´AZIENDA OSPEDALIERO-UNIVERSITARIA OSPEDALI RIUNITI DI FOGGIA |
| COMITATO ETICO DELLE AZIENDE SANITARIE DELL' UMBRIA DI PERUGIA |
| COMITATO ETICO DELL'AZIENDA OSPEDALIERA S. GERARDO DI MONZA (MB) |
| COMITATO ETICO DELL'AZIENDA OSPEDALIERA UNIVERSITARIA MATER DOMINI DI CATANZARO |
| COMITATO ETICO SCIENTIFICO DELL´AZIENDA OSPEDALIERA OSPEDALE S. CARLO BORROMEO DI MILANO |
| COMITATO ETICO DELLA AZIENDA SANITARIA PROVINCIALE DI MESSINA |
| COMITATO ETICO INDIPENDENTE DELL´IRCCS CENTRO DI RIFERIMENTO ONCOLOGICO DI AVIANO (PN) |
| COMITATO ETICO PER LA SPERIMENTAZIONE CLINICA DELLA PROVINCIA DI PADOVA |
| COMITATO ETICO DELLA ASL DI NUORO |
| COMITATO ETICO DELLA ASL RM/C DI ROMA |
| COMITATO ETICO DELL´OSPEDALE ISRAELITICO DI ROMA |
| SOTTOCOMITATO ETICO PER LA SPERIMENTAZIONE CLINICA DEI FARMACI DELLA AUSL 6 DI LIVORNO |
| COMITATO ETICO DELLA AUSL RM/H DI ALBANO LAZIALE |
| COMITATO ETICO INTERZONALE DELLA ASUR ZONA TERRITORIALE 8 DI CIVITANOVA MARCHE E ZONA TERRITORIALE 9 DI MACERATA |
| COMITATO ETICO DELL´IRCCS FONDAZIONE S. RAFFAELE DEL MONTE TABOR DI MILANO |
| COMITATO ETICO DELL´AZIENDA OSPEDALIERA S. CAMILLO - FORLANINI DI ROMA |
| COMITATO ETICO DELL´IRCCS ISTITUTO CLINICO HUMANITAS DI ROZZANO (MI) |
| COMITATO ETICO AZIENDALE DELL´AUSL 5 SPEZZINO DI LA SPEZIA |
| COMITATO ETICO LOCALE DELL´AZIENDA OSPEDALIERO-UNIVERSITARIA CAREGGI DI FIRENZE |
| COMITATO ETICO DELL´AZIENDA OSPEDALIERA SPEDALI CIVILI DI BRESCIA |
| COMITATO ETICO DELL´AZIENDA OSPEDALIERA DI DESENZANO DEL GARDA (BS) |
| COMITATO ETICO DELLA ASL RM/B DI ROMA |
| COMITATO ETICO PER LE ATTIVITA' BIOMEDICHE DELL'UNIVERSITA' DEGLI STUDI FEDERICO II DI NAPOLI |
| COMITATO ETICO DELL´AZIENDA OSPEDALIERA OSPEDALE MAGGIORE DI CREMA (CR) |
| COMITATO ETICO DI AREA VASTA ROMAGNA E ISTITUTO SCIENTIFICO ROMAGNOLO PER LO STUDIO E LA CURA DEI TUMORI DI MELDOLA (FC) |
| COMITATO ETICO DELLA ASL NAPOLI 3 SUD DI BRUSCIANO (NA) |
| COMITATO ETICO DELL´AZIENDA OSPEDALIERA UNIVERSITARIA MAGGIORE DELLA CARITA´ DI NOVARA |
| COMITATO ETICO DELLA PROVINCIA DI MODENA |
| COMITATO ETICO DELLA AUSL 3 DI CATANIA |
| COMITATO ETICO DELL' AZIENDA POLICLINICO UMBERTO I DI ROMA |
| COMITATO ETICO UNICO PER LA PROVINCIA DI PARMA |
| COMITATO ETICO DELLA ASL NA/2 NORD |
| COMITATO ETICO DELLA ASL T0/2 DI TORINO |
| COMITATO DI BIOETICA DELLA FONDAZIONE IRCCS POLICLINICO S. MATTEO DI PAVIA |
| COMITATO ETICO DELL´AZIENDA OSPEDALIERA OSPEDALE DI LECCO |
| COMITATO ETICO DELL'UNIVERSITA' DEGLI STUDI GABRIELE D`ANNUNZIO E DELLA ASL 2 LANCIANO-VASTO-CHIETI DI CHIETI |
| COMITATO ETICO DELL' AZIENDA OSPEDALIERA DI COSENZA |
| COMITATO ETICO DELLA AUSL DI BOLOGNA |
