# Supplementary material for: Classification of adults suffering from typical gastroesophageal reflux disease symptoms: contribution of latent class analysis in a European observational study
Source: BMC Gastroenterol. 2014 Jun 26;14:112. doi: 10.1186/1471-230X-14-112 (PMC4094535; doi:10.1186/1471-230X-14-112)
Supplement: Additional file 2 — Results. [file 1471-230X-14-112-S2.docx]

**Additional file 2**

**RESULTS**

***Participants***

The enrollment per country is presented in Table A. Tables B and C show a summary of the demographic characteristics and concurrent comorbidities.

A history of GERD was known in 74.3% of the participating subjects and for 66% of them, the diagnosis was established between 0 and 4 years ago. Of all subjects, 59.2% had undergone at least one upper digestive endoscopy, mainly resulting in the diagnosis of hiatus hernia (57.1%) and esophageal mucosa lesion (56.2%). In the latter group, if esophagitis was diagnosed (92.8%), the severity of lesion, according to the Los Angeles classification, was mild to moderate in 90.8% of the patients.

GERD non-medical treatment (51.4% of subjects) consisted in a continuation of the subjects’ lifestyle habits or the start with new lifestyle habits (38.9%). Overall, 66.1% received medical GERD treatment at the time of the study, with an average treatment duration of 30.5 months (SD=40.17 months). The most commonly used GERD medical treatment was PPI treatment (76.3% of subjects who received medical treatment), followed by antacids and alginate-based formulations (32.1%), and prokinetics (15.6%). H2 blockers were used by only 6.8% of the subjects receiving medical treatment for GERD. On average, PPIs had been used for more than two years (28 months). Overall, 72.5% of the subjects confirmed to continue their present medical treatment.

The severity of the typical GERD symptoms was mostly reported as moderate heartburn (53.9%) and mild regurgitation (37.1%). Almost 40% of the subjects experienced these typical GERD symptoms during 2 to 3 days per week. A frequency of 4 to 5 days per week was seen in 28.3% of the subjects, and 6 to 7 days in 22.0%. Half of the subjects suffered the typical GERD symptoms both during night and day; 38.5% of them only during the day and 11.5% only during the night. Most subjects (43.3%) indicated that their typical GERD symptoms worsened or didn’t change. A global improvement in the last 12 months was reported by 13.4% of the subjects.

Table D presents the occurrence of atypical GERD symptoms. Atypical digestive symptoms were reported by 79.9% of the subjects, whereas atypical non-digestive ENT-related symptoms occurred in 24.7% of the subjects.

Warning signs have been captured for 24.8% of the participating subjects in the week prior to the study visit (Table E).

Quality of sleep is presented in Table F. Of all subjects, 48.3% considered their sleep quality as good. However, 58.1% of the total subject population had a sleep disorder and 3.2% of the subjects were diagnosed with sleep apnea syndrome. Most (60.7%) of these sleep disturbances were considered completely or partially related to GERD.

***Association demographic data-latent classes***

Based on the subject’s history data, the highest occurrences of medical history or concurrent comorbidities were found in class 1 (Table G). Most subjects per class, who reported to smoke, drink alcohol, or adopted a lifestyle habit were found in class 1 (31.0, 41.5, and 52.8%, respectively). At least 60% of the subjects who underwent an endoscopy had a diagnosis of hiatus hernia in classes 1, 2, and 3 and 48.5 to 60.2% (over all classes) were diagnosed with esophageal mucosa lesions. The most frequently used GERD medications over all classes, were PPIs (ranging from 75.2% in class 2 to 82.2% in class 4).

**Tables**

**Table A. Number of included subjects by country**

| **Analysis, n (%)** | **France** | **Greece** | **Italy** | **Russia** | **Spain** | **Total** |
| --- | --- | --- | --- | --- | --- | --- |
| Per-protocol | 3503 (45.5) | 847 (11.0) | 1390 (18.1) | 1149 (14.9) | 811 (10.5) | 7700 (100) |
| Primary analysis | 3373 (45.4) | 806 (10.8) | 1352 (18.2) | 1108 (14.9) | 795 (10.7) | 7434 (100) |

n=number of subjects with observations

**Table B. Demographic data, overall (per-protocol analysis set N=7700)**

| **Demographic data** |  |  |  |
| --- | --- | --- | --- |
| Gender, n (%) | **Female**  4005 (52.0) | **Male**  3694 (48.0) |  |
|  | **Mean (SD)** | **Median** | **Range** |
| Age, years | 52.0 (14.9) | 52.0 | 18-98 |
| Waist, cm | 92.5 (14.0) | 92.0 | 36-193 |
| Height, cm | 168.4 (9.1) | 168.0 | 136-201 |
| Weight, kg | 76.4 (15.9) | 75.0 | 30-182 |
| Weight change since 24 months, kg* | 0.4 (5.37) | 0.0 | -50 - 41 |
| Weight change since 12 months, kg** | 0.0 (3.93) | 0.0 | -33 - 26 |
| BMI, kg/m^2^ | 26.9 (5.0) | 26.2 | 13-61 |
| 3 categories, n (%) | **Lean**  3008 (39.1) | **Overweight**  2875 (37.3) | **Obese**  1817 (23.6) |
|  | **Yes** | **No** |  |
| Smoke, n (%) | 2041 (26.5) | 5656 (73.5) |  |
| Alcohol, n (%) | 2946 (38.3) | 4750 (61.7) |  |
| Lifestyle habits, n (%) | 3429 (44.6) | 4267 (55.4) |  |
| Concomitant medications, n (%)  most commonly used  (at least 6.0% occurrence), n (%): | 4445 (57.8) | 3250 (42.2) |  |
| Other | 2674 (34.7) |  |  |
| Statins | 1321 (17.2) |  |  |
| Neuroleptics | 680 (8.8) |  |  |
| Sleeping drugs | 617 (8.0) |  |  |
| Analgesics | 604 (7.8) |  |  |
| Aspirin (low dose) | 586 (7.6) |  |  |
| Ca antagonists | 511 (6.6) |  |  |
| NSAIDS | 460 (6.0) |  |  |

SD = standard deviation

N=number of subjects

n=number of subjects with observations

* N = 5608

** N = 6493

**Table C. Concurrent comorbidities, overall (per-protocol analysis set N=7700)**

| **Most common comorbidities** (at least 5.0% occurrence) | **n** |
| --- | --- |
| Central obesity | 3740 |
| Cardiovascular disease | 2098 |
| Metabolic syndrome | 1200 |
| *Hypertension* | 968 |
| *Elevated triglycerides* | 746 |
| *Decreased HDL* | 612 |
| *Fasting plasma hyperglycemia or DM type 2* | 560 |
| Irritable bowel disease | 1062 |
| Osteoarthropathic treatment | 930 |
| Diabetes | 563 |

n=number of subjects with observations

N=number of subjects

HDL = high density lipoproteins

DM = diabetes mellitus

**Table D. Atypical GERD symptoms, overall (per-protocol analysis set N=7700)**

| **Atypical digestive symptoms** | **n (%)** |
| --- | --- |
| Epigastralgia | 3291 (42.7) |
| Eructation | 3274 (42.5) |
| Slow digestion/early satiety | 2875 (37.3) |
| Nausea | 1457 (18.9) |
| Other digestive symptoms | 799 (10.4) |
| **Atypical non-digestive symptoms** | **n (%)** |
| ENT symptoms (hoarseness pharyngeal pain globus, etc.) | 1902 (24.7) |
| Pulmonary symptoms (cough, etc.) | 1626 (21.1) |
| Thoracic manifestations (atypical precordial pain) | 1193 (15.5) |

N=number of subjects

n=number of subjects with observations

ENT = ears, nose, throat

**Table E. Warning signs**

| n (%) | **Subjects for whom warning signs are applicable (N=1909)** | **Overall (N=7700)** |
| --- | --- | --- |
| Dysphagia | 998 (52.3) | 999 (13.0) |
| Unusual (or excessive) asthenia | 579 (30.3) | 579 (7.5) |
| Vomiting | 363 (19.0) | 363 (4.7) |
| Anorexia | 307 (16.1) | 307 (4.0) |
| Weight loss | 204 (10.7) | 204 (2.6) |
| Anemia | 103 (5.4) | 103 (1.3) |
| GI bleeding | 29 (1.5) | 29 (0.4) |

N=number of subjects

n=number of subjects with observations

GI=gastrointestinal tract

**Table F. Quality of sleep**

| n (%) | **Subjects with a sleep disorder (N=4326)** | **Overall (N=7700)** |
| --- | --- | --- |
| Nocturnal awakening(s) | 2698 (62.4) | 2698 (36.2) |
| Difficulty to fall asleep | 998 (52.3) | 1407 (18.9) |
| Feeling of a bad night sleep | 1693 (39.1) | 1694 (22.7) |
| Early awakening(s) | 935 (21.6) | 935 (12.6) |
| Nightmares | 335 (7.7) | 335 (4.5) |

N=number of subjects

n=number of subjects with observations

**Table G. Medical history and concurrent comorbidities (primary efficacy set N=7434)**

| n (%) | **Class 1**  **N=1598** | **Class 2**  **N=845** | **Class 3**  **N=2375** | **Class 4**  **N=1181** | **Class 5**  **N=1435** |
| --- | --- | --- | --- | --- | --- |
| Treatment for diabetes | 124 (7.8) | 63 (7.5) | 171 (7.2) | 76 (6.4) | 67 (4.7) |
| Central obesity | 807 (50.6) | 359 (42.6) | 1140 (48.1) | 562 (47.8) | 667 (46.5) |
| Metabolic syndrome | 316 (19.8) | 123 (14.6) | 345 (14.5) | 140 (11.9) | 158 (11.0) |
| *Fasting plasma hyperglycemia/ DM type 2* | 149 | 62 | 154 | 60 | 67 |
| *High blood pressure/treated hypertension* | 256 | 95 | 270 | 116 | 135 |
| *(Treatment for) Decreased HDL* | 164 | 55 | 168 | 78 | 81 |
| *(Treatment for) Elevated triglycerides* | 199 | 73 | 229 | 79 | 85 |
| Treatment for cardiovascular disease | 510 (31.9) | 229 (27.1) | 598 (25.2) | 285 (24.1) | 344 (24.0) |
| Osteoarthropatic treatment | 272 (17.0) | 114 (13.5) | 261 (11.0) | 110 (9.3) | 114 (7.9) |
| IBS | 319 (20.0) | 109 (12.9) | 285 (12.0) | 137 (11.6) | 161 (11.2) |
| Treatment with PPD | 3 (0.2) | 1 (0.1) | 2 (0.1) | 0 | 0 |
| Polysomnographic monitoring | 13 (0.8) | 4 (0.5) | 13 (0.5) | 6 (0.5) | 6 (0.4) |
| Anti-reflux surgery | 3 (0.2) | 7 (0.8) | 6 (0.3) | 4 (0.3) | 2 (0.1) |
| Bariatric surgery | 1 (0.1) | 0 | 7 (0.3) | 0 | 1 (0.1) |

N=number of subjects

n=number of subjects with observations

DM=diabetes mellitus

HDL=high-density lipoprotein

IBS=irritable bowel syndrome

PPD=positive pressure device
